# Genetic analysis of *Pinna rudis* L 1758 (Mollusca, Bivalvia, Pinnidae) in the Northwest Cabo Verde Islands (Central-East Atlantic)

**DOI:** 10.7717/peerj.18328

**Published:** 2025-01-08

**Authors:** Evandro P. Lopes, Sarah Santos, Raquel Xavier, Joana L. Santos, M. Pilar Cabezas, Fernando Sequeira, António M. Santos

**Affiliations:** 1 Instituto de Engenharias e Ciências do Mar, Universidade Técnica do Atlântico, Mindelo, São Vicente, Cabo Verde; 2 CIBIO, Centro de Investigação em Biodiversidade e Recursos Genéticos, InBIO Laboratório Associado, Universidade do Porto, Campus de Vairão, Porto, Portugal; 3 BIOPOLIS Program in Genomics, Biodiversity and Land Planning, Universidade do Porto, Campus de Vairão, Porto, Portugal; 4 Centre of Molecular and Environmental Biology (CBMA) and ARNET-Aquatic Research Network, Department of Biology, University of Minho, Campus de Gualtar, Braga, Portugal; 5 Institute of Science and Innovation for Bio-Sustainability (IB-S), University of Minho, Campus de Gualtar, Braga, Portugal

**Keywords:** *Pinna rudis*, Cabo Verde, Genetic analysis, mtDNA, nDNA, Macaronesia

## Abstract

The rough pen shell *Pinna rudis* Linnaeus, 1758 (family Pinnidae) is a mollusc with an Atlantic–Mediterranean distribution, typically inhabiting coarse sandy substrates. Habitat degradation is considered the primary cause of population decline, leading to the designation ‘Vulnerable’ in certain regions. In this study, we conducted a genetic analysis of populations of *P. rudis* from Cabo Verde and compared them with populations from the Mediterranean and Macaronesia. We based our analysis on two mitochondrial DNA markers, cytochrome oxidase I (COI) and 16S rRNA, and one nuclear marker, 28S rRNA. The results showed a strong genetic structuring among Macaronesia populations, with each island tending to have unique or exclusive haplotypes, though some sharing occurred between islands. We found significant genetic divergence between the population from Cabo Verde and the other sampled population, suggesting that *P. rudis* is not monotypic, but may include several cryptic species. Bayesian and maximum-likelihood phylogenetic analysis, including all Pinnidae species, indicated that *P. rudis* from Gorée Island (Senegal) and Baía das Gatas (São Vicente Island) might be undergoing speciation. The high genetic structure found for *P. rudis* could be influenced by hydrodynamic barriers, local currents and hydrographic isolation, in association with the short larval duration (planktotrophic) reported for this species. Altogether, our findings highlight significant genetic divergence in *P. rudis* populations, possibly supporting speciation events in the Cabo Verde archipelago among widely distributed taxonomic groups.

## Introduction

The study of genetic diversity and evolutionary dynamics of species and populations in oceanic islands is fundamental for understanding their capacity for local adaptation, response to environmental changes and long-term survival (Carson, 1983). Sea-level fluctuations can cause major extinctions or promote population expansion (Cunha et al., 2011), and in some cases, these changes may result in the emergence of new taxonomic groups (Booy et al., 2000; Zhong et al., 2016). Identifying genetic structure within populations, often resulting from habitat fragmentation and reduced gene flow, is a critical aspect of implementing effective conservation measures to preserve the genetic diversity of distinct genetic units (Lemer et al., 2014).

Speciation in volcanic islands often occurs through one or a few inter-island founders (Carson, 1983), or through connectivity among islands. The relationship between an island’s geological history and its biodiversity has intensified the research interest in oceanic archipelagos, focusing on taxonomy, systematics, phylogeny, biogeographic patterns and species evolution. In addition, these studies often serve to outline more specific management and conservation strategies for natural populations (Marko, 2004; Wares & Cunningham, 2005). These aspects are of particular relevance in the Cabo Verde archipelago, where the geological history has played a significant role in shaping biodiversity.

In the Cabo Verde archipelago, phylogenetic studies have addressed the evolution of some marine species, primarily using mtDNA data. Among these, the gastropod *Conus* L, 1758, has been well-studied and serves as a notable example of marine diversity in this insular environment (Cunha et al., 2005, 2008, 2014; Cunha, Grande & Zardoya, 2009). However, despite bivalves being the second most common group of molluscs found in the islands, their diversity remains largely unstudied. Notably, mostly sessile species like *Pinna rudis* L, 1758, (Family Pinnidae) and *Brachidontes puniceus* Gmelin, 1791, (Mytilidae) are widely reported across the islands, yet genetic studies of these species have been scarce (Cunha et al., 2011). For sessile organisms, habitat degradation can threaten survival by causing population fragmentation, and potentially result in the emergence of new species (Basso et al., 2015; Whittaker & Fernández-Palacios, 2007*)*.

**Figure 1:**
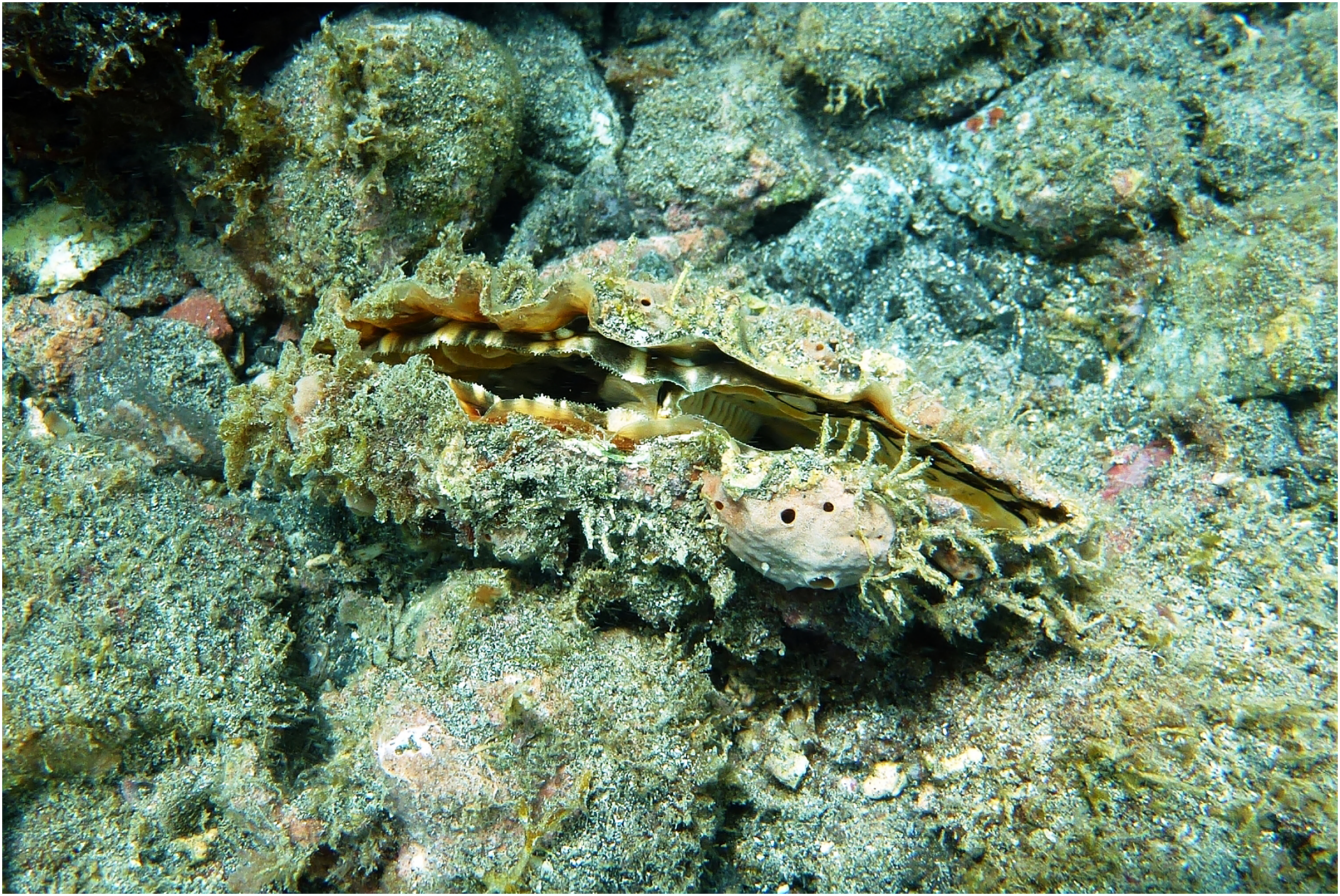
Specimen of *Pinna rudis* in its natural habitat, observed in Porto Grande Bay, São Vicente, Cabo Verde.

*P. rudis* (Fig. 1) is a widely distributed bivalve, found mainly in the Mediterranean (Zotou et al., 2023), North Atlantic (archipelagos of Azores, Madeira, Canary Islands and Cabo Verde), extending as far west as the Caribbean (Miloslavich et al., 2010) and as far south as Saint Helena and the Gulf of Guinea (Barea-Azcón, Ballesteros-Duperón & Moreno, 2008; WoRMS, 2022). It inhabits small sandy spots on rocky bottoms, and rock crevices at depths ranging from 0 m up to 60 m (García-March & Kersting, 2006; Sempere et al., 2006). The species is characterized by a shell reaching 25–30 cm, five to ten radial ribs and large spines, and vary in color (from brown to pink-orange). Like its sister species *P. nobilis* L, 1758, it is believed that *P. rudis* is a sequential hermaphrodite with asynchronous gamete maturation to avoid self-fertilization. Although there is scarce information about its planktonic larval stage, it is reported to last 5 to 10 days in the water column (Sanna et al., 2013). Such duration likely limits larval dispersal capacity and consequently reduces gene flow between distant populations.

Information about *P. rudis* populations, including their biology, ecology and distribution is still limited (Gvozdenović et al., 2019). However, *P. rudis* plays an important ecological role as a filter-feeder and is considered a good bio-indicator of environmental pollution, being more abundant in less degraded areas or with less intense anthropic influence. It also contributes to local biodiversity by providing a hard substrate for colonization of other benthic species (flora and fauna) (Basso et al., 2015; Lopes, Monteiro & Santos, 2020). Despite its ecological importance, molecular studies on *P. rudis* are rare. While mitochondrial DNA data have been published for some congeneric taxa (just “congeners” can do as well), *e.g*., *P. bicolor* and *P. nobilis* (Katsares et al., 2008; Catanese, Coupé & Bunet, 2022), studies of *P. rudis* remain limited.

Classification of taxa in Pinnidae is challenging due to the variability in morphological characteristics and the lack of consistent morphological data for reliable identification (Bieler & Mikkelsen, 2006). Genetic data suggest that the family may include cryptic species (Lemer et al., 2014), as the plasticity of the shell renders it of limited taxonomic value. Currently, *Pinna* species are often identified based on morphological characteristics, which is particularly challenging at the juvenile stage. While *Pinna* species are present in Cabo Verde, no genetic studies have been conducted in the region. As genomic data become more accessible, integrating diverse data types and improving analytical models will be crucial for accurate taxonomic identification, particularly for cryptic species. This knowledge is ultimately crucial for advancing biodiversity conservation efforts. For example, a pilot study in the Mediterranean, using genetic information, enabled the identification of hybrids between *P. rudis* and *P. nobilis*, which may have contributed to saving *P. nobilis* from extinction by introducing new variants in the genetic pool (Vázquez-Luis et al., 2021).

Given the wide distribution of *P. rudis* and its short larval stage, genetic structuring within Macaronesian populations is expected. Previous studies on molluscs have shown that long distances between suitable substrates and oceanographic barriers, such as currents and deep areas, between the islands may have driven the adaptive radiation of some mollusc species in Cabo Verde, *e.g*., *Conus* (Cunha et al., 2005, 2008, 2014; Cunha, Grande & Zardoya, 2009; Duda & Lessios, 2009; Duda & Rolán, 2005; Puillandre et al., 2014; Tenorio, Afonso & Rolán, 2008; Tenorio & Afonso, 2004), *Fissurella* (Cunha et al., 2017), *Euthria* (Fraussen & Swinnen, 2016) and Nudibranchia gastropods (Calado, Ortea & Caballer, 2005; Ortea-Rato & Espinosa, 1998; Ortea, 1989; Pola et al., 2014; Rolán, 2005; Wirtz, 2009).

To better understand the evolutionary history of *P. rudis*, its current conservation status, and its potential resilience to ongoing habitat loss, it is essential to investigate its genetic diversity. It is against this background that the present study aims to improve our understanding of the genetic differentiation between populations of *P. rudis* in Cabo Verde, by combining genetic and morphological data. Additionally, the study seeks to reconstruct phylogenetic relationships between *P. rudis* populations from the northeast Atlantic, Mediterranean and Macaronesian regions. To this end, we sequenced portions of two mitochondrial markers, cytochrome C oxidase subunit I (COI) and 16S rRNA, as well as one nuclear marker, 28S ribosomal rRNA.

The findings of this study have important implications for the conservation and management of *P. rudis*, particularly in regions facing increasing human pressure. Understanding the genetic structure and potential cryptic diversity within these populations is essential to develop effective conservation strategies and to preserve the species and the unique genetic heritage they represent. Moreover, this research may contribute to our understanding of the influence of isolation and environmental heterogeneity on genetic diversity and the phylogenetic relationships in marine invertebrates.

## Methodology

### Study area

**Figure 2:**
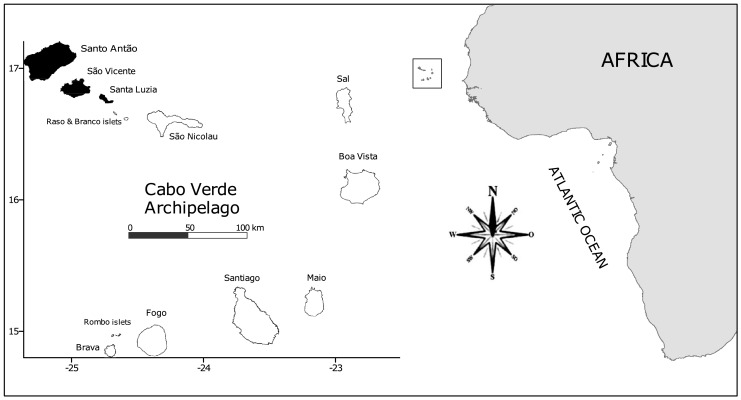
Location of the Cabo Verde Archipelago and the study islands in black (Santo Antão, São Vicente and Santa Luzia).

Cabo Verde is an archipelago located 600 km from the West African coast (Fig. 2), at 14°50′-17°20′N and 22°40′-25°30′W. It comprises 10 volcanic islands, ranging in age from approximately 3 to 158 Mya. The archipelago is divided into three basic groups: the northwestern group (Santo Antão, São Vicente, Santa Luzia and São Nicolau), the southern group (Santiago, Fogo and Brava), and the eastern group (Sal, Boavista and Maio). Boavista Island is situated about 570 km off Senegal, on the west African coast. Cabo Verde has a coastline of ca. 2,000 km, and a land area of 4,033 km^2^ (Medina et al., 2007; Freitas et al., 2019).

The archipelago is characterized by a narrow shelf and deep waters, reaching depths of up to 4,000 m between the islands (Mason, Coombs & Oliveira, 2005). Oceanographically, Cabo Verde is influenced by the North Equatorial counter-current and the Canary Current. The sea surface temperatures normally exceed 20 °C throughout the year. Together with the Canary Islands, Azores and Madeira, Cabo Verde forms part of the biogeographical region known as Macaronesia (Peña-Izquierdo et al., 2012). However, long-term data and more refined analysis suggest that Cabo Verde has unique biogeographic characteristics (Freitas et al., 2019*)*. Studies on certain marine groups in Cabo Verde have even suggested the exclusion of this archipelago from Macaronesia, instead classifying it as part of a West African biogeographic sub-province (Freitas et al., 2019).

### Sample collection and processing

Data collection was performed by SCUBA-diving at 0.5–4 m depth. Samples were collected at the following sites: Santo Antão-Porto Novo (17°0′4604″N/25°5′579″W), São Vicente–Laginha (16°53′5506″N/24°59′3634″W) and Baía das Gatas (16°54′755″N/24°54′2168″W); and Santa Luzia Islands–Portinho (16°45′945″N/24°45′3073″W), under permission from Cabo Verde government (permits DNA_N°08/2017). *P. rudis* specimens collected (*n* = 20) were photographed using a Panasonic Lumix DMC-TZ20 digital camera. The samples were transported to the ISECMAR-UTA Molecular Biology Laboratory and kept in the cold freezer (−28 °C) in separate bags. Initially, the goal was to sample approximately 10 specimens per site. However, due to very low concentrations of the species in some places and considering the conservation status of the species (which is included in Annex II of the Bern Convention as strictly protected species, and in the Barcelona Convention as threatened or endangered marine species) (Nebot-Colomer et al., 2016), it was not possible to collect that number for some localities. The specimens used for genetic analysis were deposited in the collection of the Universidade Técnica do Atlântico, São Vicente, Cabo Verde.

### Morphological analyses

Morphological characters were selected based on the taxonomic descriptions in Poutiers (2016) and Abbot & Dance (2000). The biometric data obtained from 32 shell samples, resulted in a data matrix of five parameters: maximum shell length-L, maximum shell width-Lt, total shell weight W, L/W ratio, and Lt/W ratio (Supplemental Material, Table S1). We investigated the morphological variation within *P. rudis* across the different Cabo Verde sampling sites. Statistical differences in the mean sizes among the four groups were analysed using a one-way ANOVA, followed by a Tukey’s honestly significant difference (HSD) *post hoc* test. The level of significant difference was at *p* < 0.05. Principal components analysis (PCA) was conducted on log-transforming shell measurements to identify morphometric characters with the most variation among populations. Finally, a discriminant analysis (DA) was conducted to differentiate the meristic parameters of the four populations. All morphological analyses were performed using PAST3 software (Hammer, Harper & Ryan, 2001).

### DNA isolation and sequencing

DNA extraction was performed from a portion of the posterior adductor muscle. Tissue was taken from the inside of the muscle to avoid polysaccharides that may reduce the efficiency of DNA extraction (Panova et al., 2016). Tissue samples were stored in a 2-ml Eppendorf tube with 96% alcohol at −21 °C until DNA extraction. Genomic DNA was extracted following Sambrook & Russel (2001) using a sodium chloride protocol. Muscle tissue (100–250 mg) was excised and homogenized in extraction buffer (0.05 M Tris, 0.1 M EDTA, pH 7) with 70 µl of SDS and 14 µl proteinase K (10 ng per µl). The homogenates were incubated at 55 °C for 4 h, and DNA was precipitated with isopropanol. DNA pellets were washed with 70% ethanol, dried and suspended in 100 µl of water. Extraction products were visualized using 1% agarose gel electrophoresis stained with Sybr Green. Samples were then stored at 4 °C. DNA concentration was estimated using a spectrophotometer. Samples with low DNA concentrations (*n* = 12) were reprocessed using the EZNA Tissue DNA Kit (Omega Bio-Tek) according to the manufacturer’s instructions.

Fragments of the two mitochondrial—16S (490 bp) and COI (644 bp)—and one nuclear marker—a fragment of 28S rRNA (ca. 2Kb)—were amplified using a polymerase chain reaction (PCR) with primers showing a certain level of polymorphism within the used specimens, according to Lemer et al. (2014, 2016) and Vázquez-Luis et al. (2021). (See Table 1 for complete primer information). In some cases the primers used to amplify the COI fragment were unsuccessful, probably due to the presence of polysaccharides (Bessetti, 2007). Therefore, a second primer pair jgHCO-2198 and jgLCO-1490 (Geller et al., 2013) was used for those cases. PCR was performed in a 25-µL solution containing: 1X PCR buffer, 25 mM of dNTP, 0.5 mM of each primer, 2.5 mM of MgCl2, 2 μl of template DNA, 01 μg/μl BSA and 0.3 U of Platinum Taq DNA Polymerase (Invitrogen, Carlsbad, CA, USA). Reactions were carried out using the following cycling parameters: initial denaturing at 94 °C for 5 min, 45 cycles of 94 °C for 45 s, 48–50 °C (16S rRNA), 48–55 °C (COI), or 52 °C (28S rRNA), for 1 min, and 72 °C for 90 s and a final extension at 72 °C for 10 min double-stranded.

**Table 1:** Primers sequences used in this study and the annealing temperature at which successful amplifications were performed.

Gene	Primer name	Sequence (5′ – 3′)	Annealing temperature (°C)	Source
16S	16Sar	CGCCTGTTTATCAAAAACAT	48–50	Palumbi (1996)
	16Sbr	CCGGTCTGAACTCAGATCACGT	48–50	Palumbi (1996)
COI	HCO-2198	TAAACTTCAGGGTGACCAAAAAATCA	48–49	Folmer et al. (1994)
	LCO-1490	GGTCAACAAATCATAAAGATATTGGGG	48–49	Folmer et al. (1994)
	jgHCO2198	TAIACYTCIGGRTGICCRAARAAYCA	50–55	Geller et al. (2013)
	jgLCO1490	TITCIACIAAYCAYAARGAYATTGG	50–55	Geller et al. (2013)
28S	28Sa	GAC CCGTCTTGAAACACGGA	52	Whiting et al. (1997)
	28Srdb	CCACAGCGCCAGTTCTGCTTAC	52	Whiting et al. (1997)
	28Srd1a	CCCSCGTAAYTTAGG CATAT	52	Edgecombe & Giribet (2006)
	28Srd4b	CCTTGGTCC GTG TTTCAAGAC	52	Edgecombe & Giribet (2006)
	28Srd4.8a	ACCTATTCTCAAACTTTA ATGG	52	Schwendinger & Giribet (2005)
	28Srd7b1	GACTTCCCTTAC TACAT	52	Schwendinger & Giribet (2005)

PCR products were visualized using 1% agarose gel electrophoresis stained with Sybr Green. Approximately 10 ng of the purified product was used as a template for sequencing, performed in an automated sequencer (ABI PRISM 3700; Applied Biosystems, Foster City, CA, USA) using the BigDye® Terminator v31 Cycle Sequencing Kit (Applied Biosystems, Foster City, CA, USA), following the manufacturer’s instructions.

### Sequence data

A manual check of misreads in COI, 16S rRNA and 28S rRNA chromatograms was performed with Bioedit v7053 for Windows (Hall, 1999). Taxonomic confirmation was done through a BLAST search (National Center for Biotechnology Information, 2023). The translation of COI was inferred using the invertebrate mtDNA genetic code in Unipro UGENE version 129 (Okonechnikov et al., 2012*)*. Multiple sequence alignments were performed with the Clustal W algorithm present in BioEdit, with a gap opening penalty of 10,000 and a gap extension penalty of 0,05. The alignments obtained were optimized manually when needed. Regions of poor alignment within the ribosomal RNA genes, typically unpaired loops and bulges of varying sizes bounded by stem regions, were excluded from the phylogenetic analyses using GBlocks Server v091b (Castresana, 2000). The haplotypes (mtDNA) were determined separately and from concatenated (1,073 bp) sequences from the two regions of the mitochondrial gene encoding the COI and 16S rRNA fragments.

### Phylogenetic analysis

For the phylogenetic analyses, additional COI, 16S rRNA, and 28S rRNA sequences were downloaded from GenBank for the nine species of the Pinnidae family (*P. rudis, P. carnea* Gmelin, 1791, *P. atropurpurea* G B Sowerby I, 1825, *P. bicolor* Gmelin, 1791, *P. dolabrata* Lamarck, 1819, *P. muricata* L, 1758, *P. nobilis, P. trigonalis* Pease, 1861 and *P. epica* Jousseaume, 1894) and the outgroup *Streptopinna saccata* (L, 1758). The genus *Streptopinna* was chosen as an outgroup based on its proximity to the genus *Pinna* according to Lemer et al. (2014). The sequences used in gene tree reconstruction are detailed in Table 2.

**Table 2:** Sequences of mitochondrial COI, 16S and nuclear 28S genes of Pinnidae species used in the genetic analysis. The 20 new sequences from Cabo Verde were the only ones obtained in this study and the GenBank accession numbers are presented here for each marker. The total number of specimens per species is given as well as the location where it was found and the GenBank accession numbers for each. *Streptopinna saccata* species was included in the species tree reconstruction as outgroup.

Taxon	Location 1	Location 2	Region	N	ID	Specimens	16S rRNA	COI	28S rRNA
*Pinna rudis* Linnaeus, 1774	Cabo Verde–Santo Antão	Porto Novo	Atlantic Ocean	6	VA1	PRUD (VA1, 1–6)	OQ055700–OQ055705	OQ026776 OQ026781	OQ026797 OQ026802
	Cabo Verde–São Vicente	Laginha		6	VV1	PRUD (VV1, 1–6)	OQ05576–OQ055711	OQ026782–OQ026787	OQ026803–OQ026808
		Baía das Gatas		5	VV2	PRUD (VV2, 1–5)	OQ055712–OQ055716	OQ026788–OQ026792	OQ026809–OQ026813
	Cabo Verde–Santa Luzia	Portinho		3	VL1	PRUD (VL1, 1–3)	OQ055717–OQ055719	OQ026793–OQ026795	OQ026814–OQ026816
	Spain	Murcia	Mediterranean Sea	1 1	Pr	Pr13	–	KJ366482	KJ366289
					Pr	Pr15	KJ365726	KJ366483	KJ366291
		Columbrete island	Mediterranean Sea	1	Pr	Pr44	KJ365731	–	KJ366298
	Canarias island	El Hierro	Atlantic Ocean	1	Pr	Pr24	KJ365727	KJ366484	KJ366292
				1	Pr	Pr25	KJ365728	KJ366485	KJ366293
				1	Pr	Pr28	KJ365729	KJ366486	KJ366294
				1	Pr	Pr29	–	KJ366487	KJ366295
		Tenerife	Atlantic Ocean	1	Pr	Pr40	KJ365730	–	KJ366296
				1	Pr	Pr42	–	KJ366488	KJ366297
				1	Pr	Pr57	KJ365733	KJ366489	KJ366301
				1	Pr	Pr58	KJ365734	KJ366490	KJ366302
				1	Pr	Pr59	–	–	KJ366303
		Fuerteventura	Atlantic Ocean	1	Pr	Pr56	KJ365732	–	KJ366300
	Azores	São Miguel	Atlantic Ocean	1	AZ1	MZ USP 114025	KJ365520	KJ366319	KJ366026
				1	AZ2	MZ USP 114038-1	KJ365521	KJ366320	KJ366027
				1	AZ3	MZ USP 114038-2	KJ365522	KJ366321	KJ366028
	Senegal	Gorée island	Atlantic Ocean	1	SN1	MNHN IM-2013-7009	KJ365642	–	KJ366172
				1	SN2	MNHN IM-2013-7010	–	–	KJ366181
*Pinna carnea* Gmelin, 1791	Panama	Bocas del Toro	Atlantic Ocean	1	PA1	MCZ MAL-381150	KJ365533	KJ366336	KJ366044
	Florida	Long Key Channe	Atlantic Ocean	1	FL1	UF 437518	KJ365608	KJ366396	KJ366126
	French Antilles	Guadeloupe	Atlantic Ocean	1	FA1	MNHN IM-2013-7108	KJ365634	KJ366408	KJ366157
*Pinna atropurpurea* Sowerby I, 1825	Japan	Okinawa	Pacific Ocean	1	JA1	UF 351968C	KJ365595	–	KJ366112
	Philippines	Mactan island	Pacific Ocean	1	PH1	FLMNH_MO_349489	KJ365632	–	KJ366154
				1	PH2	MCZ MAL-381081	KJ365564	KU987199	KJ366080
*Pinna bicolor* Gmelin, 1791	Oman	Bar Al Hikman Peninsula	Red Sea	1	OM1	UF_367994	KJ365599	–	KJ366116
	Mozambique	Inhaca island	Indian Ocean	1	MO1	MNHN IM-2013-7077	KJ365682	–	KJ366225
			Indian Ocean	1	MO2	MNHN IM-2013-7078	KJ365691	–	KJ366234
	China	southern China coast	Pacific Ocean	1	PB	PB1		JN182725	
*Pinna dolabrata* Lamarck, 1819	Australia	Woody island	Pacific Ocean	1	AU1	MNHN IM-2013-7088	KJ365700	–	KJ366246
				1	AU2	MNHN IM-2013-7087			KJ366236
		Heron island	Pacific Ocean	1	AU3	UF 437384	KJ365590	–	KJ366107
*Pinna muricata* Linnaeus, 1758	Madagascar	Nosy be	Indian Ocean	1	MA1	UF 423478_a	KJ365579	KJ366375	KJ366095
	Djibouti		Red Sea	1	DJ1	UF 455816	KJ365585	KJ366381	KJ366102
	Guam	Pago bay	Pacific Ocean	1	GU1	MO_ 298856	KJ365627	KJ366402	KJ366148
*Pinna nobilis* Linnaeus, 1758	Spain	Columbretes	Mediterranean Sea	1	Pn	Pn8	KJ365725	–	KJ366286
		Palma de Mallorca	Mediterranean Sea	1	Pn	Pn68	KJ365721	–	KJ366281
				1	ML4	mallorca4	–	KY321774	–
		Múrcia	Mediterranean Sea	1	ML16	murcia16	–	KY321811	–
*Pinna trigonalis* W. H. Pease, 1861	French Polynesia	Marquesas islands	Pacific Ocean	1	FP1	MNHN IM-2013-7035	KJ365661	KJ366434	KJ366198
				1	FP2	MNHN IM-2013-7038	KJ365687	KJ366457	KJ366230
				1	FP3	MNHN IM-2013-7045	KJ365679	KJ366451	KJ366221
*Pinna (Abyssopinna) epica* Jousseaume, 1894	Papua New Guinea		Pacific Ocean	1	PG1	MNHN IM-2013-7084	KJ365667	KJ366440	KJ366205
	New Caledonia	South of Grande Terre		1	nC1	MNHN IM-2013-7116	KJ365639	KJ366414	KJ366165
	New Caledonia	North of Grande Terre		1	nC2	MNHN IM-2013-7023	KJ365694	KJ366462	KJ366239
*Streptopinna saccata* (Linnaeus, 1758)	Hawaii	French Frigate Shoals	Pacific Ocean	1	Ssaca1	UF 413844	KJ365601	KJ366391	KJ366119
	French Polynesia	Tahiti		1	Ssaca2	MCZ MAL-381010	KJ365742	KJ366495	KJ366314

A total of three databases were created for each marker, including the sequences from Genbank. To compare the relationships of *P. rudis* haplotypes between the Atlantic and Mediterranean, maximum parsimony networks (MP) analyses of mtDNA and nDNA were performed independently, using the statistical parsimony procedure in TCS121 (Clement, Posada & Crandall, 2000). The non-rooted haplotype networks based on the 95% parsimony were constructed separately for mitochondrial and nuclear markers. The results were visualized with tcsBU (Santos et al., 2015), treating gaps as a 5th state for the nDNA.

A second phylogenetic analysis was conducted using maximum likelihood estimation (ML), Bayesian (BI) and BEAST inference to reconstruct the phylogenetic tree of *Pinna* species. The best-fit nucleotide substitution model was identified using PartitionFinder v111 (Lanfear et al., 2012), according to Akaike’s (1973) Information Criterion. Codon partitioning was applied to COI to minimize the effects of codon position saturation (Salemi, 2009) and to account for different rates of evolution of each codon (Pond, Poon & Frost, 2009). The best common models of nucleotide substitution selected and implemented in MrBayes, RAXML and BEAST were: COI partitions: TRN + G (1st partition), GTR + X (2nd and 3rd partitions); 16S rRNA and 28S rRNA partitions: GTR + I + G (for all partitions in BEAST).

For BI analyses in MrBayes v326 software (Ronquist et al., 2012), five million generations were run with sampling every 1,000 generations, discarding the first 25% of generations as burn-in. Parameter convergence was checked using Tracer v17 (Rambaut & Drummond, 2013; Rambaut et al., 2018), ensuring ESS values > 100. A final Bayesian majority-rule consensus tree was obtained for each data set. ML analyses were performed with RAxML v8116 (Stamatakis, 2006) using a rapid hill-climbing algorithm and 10,000 bootstrap pseudoreplicates. BEAST version 2.2.1 (Bouckaert et al., 2014) was used to estimate the species tree, with a 5,000 burn-in (corresponding to 10% of the total samples in each run). Results were checked in TRACER v15 (Rambaut & Drummond, 2013) to determine adequate burn-in. The convergence and ESS were also assessed with Tracer after discarding the burn-in samples. The final species tree was plotted with Tree Annotator v245 (http://tree.bio.ed.ac.uk/software/beast/). Consensus trees were visualized in FigTree version 143 (Rambaut, 2012). Final modifications, such as the insertion of posterior values and branch colouring, were performed with Inkscape version 1.0 (https://www.inkscape.org/).

## Results

### Morphological analyses

**Figure 3:**
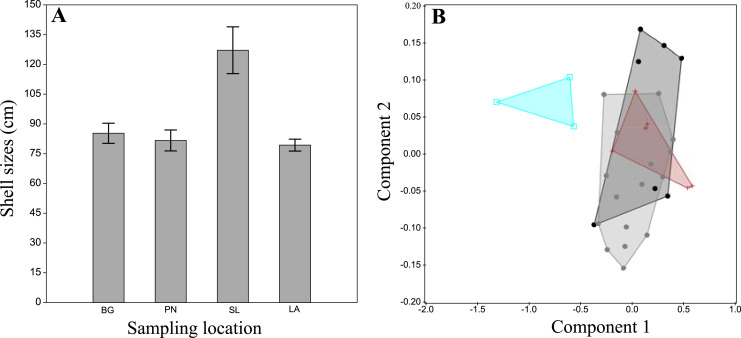
Morphological analysis of *P. rudis* specimens in the laboratory. (A) A plot of the results of average sizes (with error bar) and (B) the Principal Components Analysis result of the *Pinna rudis* specimen’s measurements from Cabo Verde. Legends: black circles-Baía das Gatas (BG), grey circles-Laginha (LA), blue squares-Portinho (PT) and red crosses-Porto Novo (PN).

The examination of morphological variation within *P. rudis* across different Cabo Verde sampling sites revealed no distinct patterns in shell morphology, colour and shape of the adductor muscle impressions. Size of the 32 specimens analysed ranged from 124.5 cm in Porto Novo to 309 cm in Portinho (Fig. 3A). Mean sizes (±SEM) were highest in Portinho (mean = 254.16 ± 27.42 cm), followed by Baía das Gatas (mean = 177.4 ± 10.50 cm), Laginha (mean = 163.09 ± 4.57 cm), and lowest in Porto Novo (mean = 158.66 ± 11.47 cm). A one-way ANOVA indicates statistically significant differences in mean size (F = 11.67, *p* < 0.005) between the Santa Luzia and others tree groups. The differences in means were not significant (*p* > 0.05) between the São Vicente and Santo Antão groups.

PCA analysis and Discriminant Analysis (DA) did not reveal any separation between the two clades of *P. rudis* from Cabo Verde sampling sites (Fig. 3B). However, a distinction was noted for samples from Santa Luzia, likely related to the overall sample size. Principal components 1 and 2 accounted for 91.03% of the variation among the samples.

### Sequenced data

A total of 65 mitochondrial DNA sequences of *P. rudis* were analysed in this study. These comprised 40 sequences generated in this study (20 COI and 20 16S rRNA sequences), and additional sequences obtained from online databases (12 COI and 13 16S rRNA sequences). For the 28S rRNA gene, 38 sequences were analysed, including 20 new sequence samples and 18 from previous studies. The final concantenated alignments for COI, 16S rRNA and 28S rRNA genes totalized 3,444 bp. No stop codons were found at least in one reading frame of the COI alignment. BLAST results confirmed the identification of *P. rudis* specimens. The sequences obtained in this study have been deposited in GenBank (OQ055700–OQ055719, OQ026776–OQ026795, OQ026797–OQ026816. See Table 2 for sequence details).

### Haplotype network reconstruction

**Figure 4:**
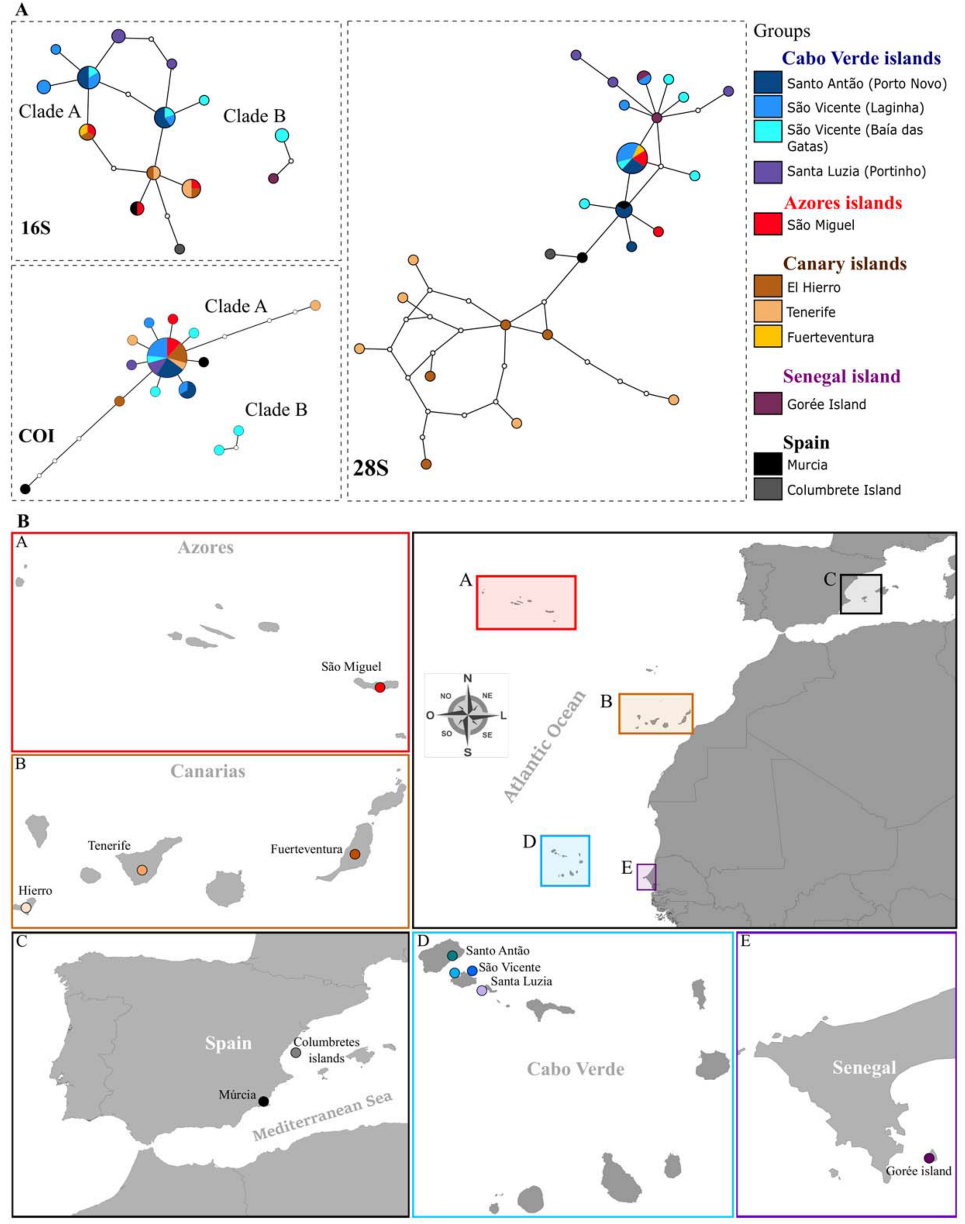
Haplotype networks and Map of sampling localities. (A) Haplotype networks (95% parsimony connection limit) for all sequenced *P. rudis* COI, 16S and 28S data. Lines connecting white dots represent mutation steps between haplotypes, while white dots represent theoretical intermediate haplotypes. (B) Map of sampling localities included in the present work: A-Azores islands, B-Canary Islands, C-Spain, D–Cabo Verde islands, E-Gorée Island.

The genetic structure of *P. rudis* populations across Macaronesia was inferred from 103 mitochondrial and nuclear DNA sequences from the archipelagos of the Azores, Madeira, Canaries, Cabo Verde and the Mediterranean (Table 2). Statistical parsimony networks for COI, 16S rRNA and 28S rRNA haplotypes are shown in Figs. 4A and 4B. Moderate diversity values were observed for both mtDNA and nDNA, with the highest diversity found in the 28S rRNA (see Table 3 for more detail).

**Table 3:** Genetic diversity indices for *Pinna rudis* in each sampling site.

Sampling places	mtDNA (16S, 470 pb)					mtDNA (COI, 594 pb)					nDNA (28S, 2,079 pb)				
	*N*	h	Hd ± SD	π ± SD	k	*N*	S	Hd ± SD	π ± SD	k	*N*	h	Hd ± SD	π ± SD	k
Cabo Verde islands	20	8	0.853 ± *0.052*	0.010 *± 0.004*	5.07	20	8	0.695 ± *0.108*	0.018 *± 0.010*	10.82	20	11	0.763 ± *0.103*	0.002 *± 0.001*	4.41
Spain	2	2	1.000 ± *0.500*	0.006 *± 0.003*	3.00	2	2	1.000 ± *0.500*	0.010 *± 0.005*	6.00	3	3	1.000 ± *0.272*	0.001 *± 0.001*	1.33
Canarias islands	7	3	0.762 ± *0.115*	0.003 *± 0.001*	1.52	7	4	0.714 ± *0.181*	0.003 *± 0.001*	1.71	10	10	1.000 ± *0.045*	0.004 *± 0.001*	5.44
Azores	3	3	1.000 ± *0.272*	0.006 *± 0.002*	2.67	3	2	0.667 ± *0.314*	0.001 *± 0.001*	0.67	3	2	0.667 ± *0.314*	0.001 *± 0.000*	1.33
Senegal	1	1	–	*-*	–	0	–	–	–	–	2	2	1.000 ± *0.500*	0.002 *± 0.001*	1.00
Total/average	33	11	0.866 ± *0.035*	0.010 *± 0.003*	4.89	32	14	0.720 ± *0.087*	0.012 *± 0.007*	7.69	38	15	0.693 ± *0.092*	0.005 *± 0.001*	2.54

**Note:**

The number of individuals used for mitochondrial and nuclear (N), number of haplotypes (h), haplotype diversity (Hd), nucleotide diversity (π), standard deviation (SD) (shown in italic), and mean of pairwise differences (k) are shown for all locality.

For COI, the species’ haplotype diversity (*h*) was 0.720 (+ 0.087), corresponding to 14 haplotypes, and nucleotide diversity (*π*) was 0.012 (+ 0.007). The COI network revealed two poorly structured networks: one star-shaped and largely shared, the other more localized, including samples from Baía das Gatas and São Vicente Islands (Fig. 4A). For 16 rRNA, haplotypic diversity was also high (*h* = 0.866 ± 0.035) and nucleotide diversity was low (π 0.010 ± 0.003). In this marker, all Cabo Verde haplotypes were unique to the archipelago, highlighting the separation of these populations from the rest. Notably, Santa Luzia’s haplotypes were not shared with any other locality, further suggesting local differentiation. Two distinct clades emerged in both mitochondrial markers, with Cabo Verde samples from Baía das Gatas and São Vicente (Fig. 4A) constituting a separate group, clustering with samples from Gorée, Senegal. Few mutational steps separated the haplotypes from other localities, with Mediterranean populations (Murcia and Columbrete Island) showing the highest divergence. The North Atlantic islands (Azores and Canary Islands) shared most haplotypes (see Figs. 4A and 4B).

For the 28S rRNA marker, the average genetic diversity values showed a slightly different pattern to that observed with the mtDNA markers (*h* = 0.693 ± 0.092; π 0.005 ± 0.001). The genetic divergence shows 26 distinct haplotypes and many private haplotypes identified. In total, 18 sites were variable with eight being parsimony informative. Samples from the Canary Islands were mostly separated from the others, although with few mutational steps between them. Interestingly, Azorean samples were genetically closer to those from Cabo Verde, despite the Canary Islands’ geographic proximity to Cabo Verde (see Figs. 4A and 4B and Table 3). Cabo Verde showed a high degree of endemic haplotypes, with only two haplotypes shared with the Azores and Murcia populations. The samples from Santa Luzia and Baía das Gatas, which showed variation in the mitochondrial networks, also highlighted differentiation in the 28S rRNA marker, though with fewer mutational steps.

### Phylogenetic relationships within *Pinna* species

Phylogenetic analyses produced trees with similar general topologies for mitochondrial data, differing only in the position of some haplotypes within internal groups, and the statistical support of clades. The 28S rRNA sequences showed a similar trend, although with low bootstrap support due to low genetic variation (*i.e*., replacement of a single nucleotide). Maximum likelihood bootstrap support (BP) and posterior Bayesian probability values (PP) were congruent with each other, with PP values generally higher, as expected (Suzuki, Glazko & Nei, 2002) (Supplemental Files, Figs. S1 and S2).

All *Pinna* species examined were monophyletic across all analyses, with high support, except for the *P. rudis* (Supplemental Files, Figs. S1 and S2) and *P. nobilis* in the mitochondrial data. The paraphyly of *P. nobilis* may result from incomplete data for the two mitochondrial markers. Both ML and PP analyses based on mitochondrial sequences showed two clades within *P. rudis* populations from Cabo Verde (Clades A and B), with bootstrap support >70%, consistent with the haplotype network reconstruction. Clade A comprised many Cabo Verde specimens grouped along with those from other Macaronesian islands and the Mediterranean, while Clade B grouped Cabo Verde samples with those from Gorée, Senegal. The average distance between the two clades was 25%, with intra-clade distances of 0.3% and 0.2%, for Clade A and Clade B, respectively. Clade B was formed by three sequences that represent two haplotypes of specimens that were collected in Baía das Gatas and on the island of Gorée, Senegal. Clade A is sister to *P. carnea* with bootstrap support >70% and Clade B to those in the mitochondrial and nuclear gene trees (Supplemental Files, Figs. S1 and S2).

**Figure 5:**
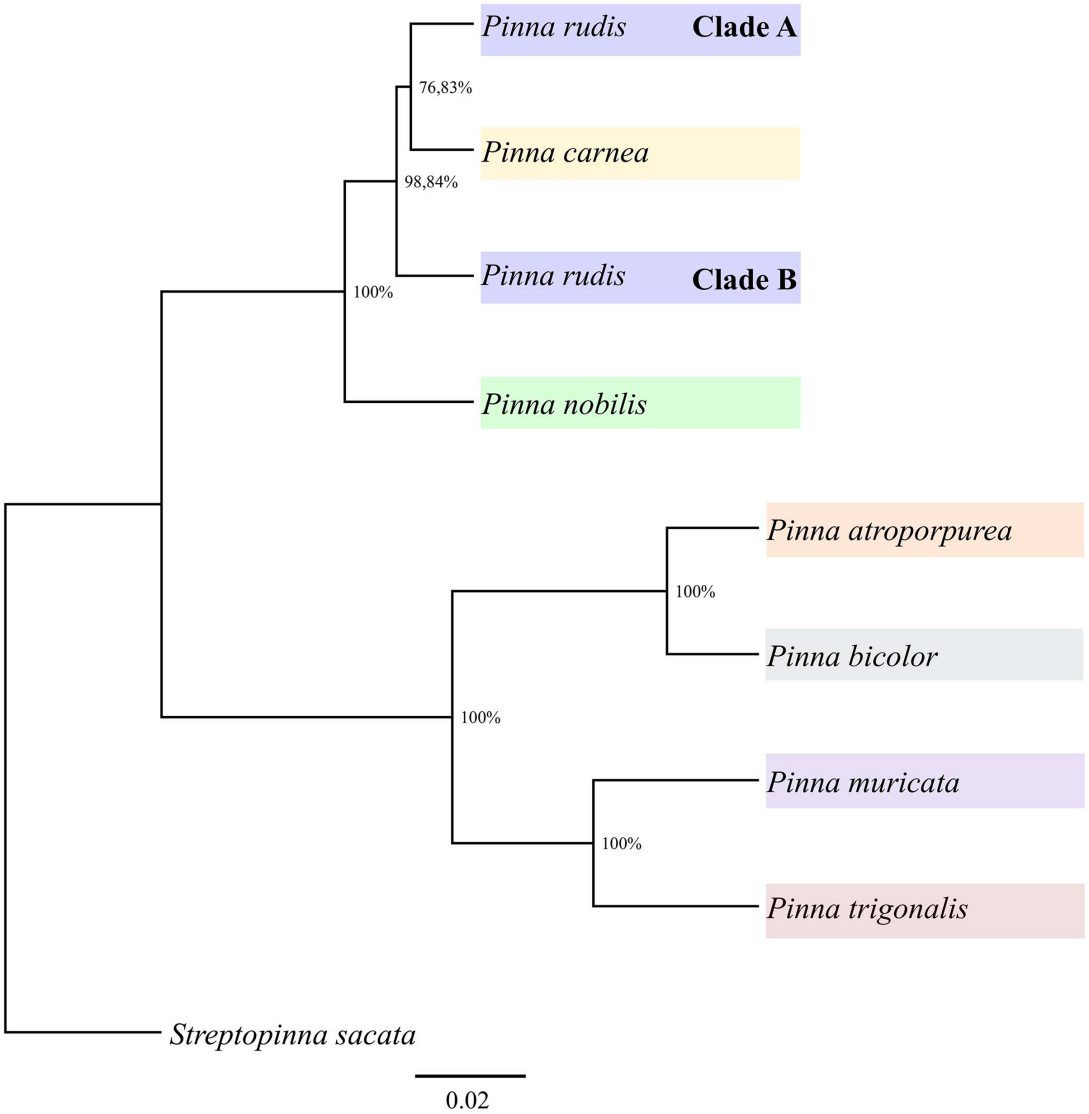
Phylogenetic relationships of Pinna genera based on a Bayesian inference analysis of mitochondrial COI, 16S rRNA and nuclear 28S rRNA markers produced by BEAST. The *Streptopinna sacata* sequence was used as an outgroup.

The BEAST analyses corroborated these findings, revealing two clades within *P. rudis* separated by *Pinna carnea* (Fig. 5), although the relationships within clades differ from the previous analysis. Specimens of *P. rudis* from Senegal and Cabo Verde (Clade B) were genetically distinct from those in the Mediterranean, Azores and Canary Islands (Clade A). Clade B shows a sister group to the remaining *P. rudis* (Clade A), *P. nobilis* and *P. carnea*.

## Discussion

This study provides a comprehensive analysis of genetic variation in *P. rudis* by combining data from both this and previous studies across the Mediterranean Sea, Macaronesia and coastal Senegal. This approach enhances our understanding of large-scale genetic patterns and allows us to revisit previous phylogenetic analyses of the *Pinna* genus conducted by Lemer et al. (2014). The haplotype network reconstructions, incorporating sequences from various regions revealed a distinct level of population structuring, that generally aligns with the geographic distribution of these populations.

The population of *P. rudis* from Cabo Verde showed great haplotypic and nucleotide diversity across all markers used. Specifically, Baía das Gatas revealed a great genetic diversity, indicating the presence of highly divergent haplotypes in sympatry. This phenomenon suggests the potential for sympatric speciation, where multiple evolutionary units coexist within the same geographical area and can interbreed. Such events will be eventually lead to the emergence of distinct species (Hart et al., 2018).

The mitochondrial markers separated two differentiated evolutionary units within Cabo Verde, demonstrating a clear separation in the haplotype network. The Baía das Gatas samples comprised unique haplotypes distinct from those in other regions. This high level of diversity is likely advantageous for population resilience, potentially detecting evolutionary processes (Zhan et al., 2021; Uricoechea Patiño et al., 2023).

The two locations in São Vicente, Baía das Gatas and Laginha, demonstrated a certain degree of haplotype sharing (two in 16S rRNA and one in 28S rRNA), likely due to their proximity. This connectivity might be influenced by the small-scale current regime around the island (Medina et al., 2007). However, the absence of the most divergent haplotype of Baía das Gatas in the population of Laginha Beach may be due to the relatively low number of samples analysed in both populations but could also suggest that ecological differences between these sites affects the distribution of evolutionary units. Baia das Gatas, characterized by its shallow lagoon behind a breakwater wall (Schönfeld & Lübbers, 2020) and limited hydrodynamism, may have historically influenced genetic connectivity. Fossil data indicating past submersion of Baia das Gatas supports the idea that historical sea level changes could have influenced current genetic diversity and connectivity (Boekschoten & Best, 1988).

Across Macaronesia and the Mediterranean, the haplotype networks showed some structuration within *P. rudis*, although mutation steps were insufficient to separate between the Micronesian archipelagos (Freitas et al., 2019), except for the unique haplotypes from Baía das Gatas (see Figs. 4A and 4B). The 16S rRNA marker again highlighted population structure, with Cabo Verde samples showing distinct clades. The most divergent samples from Baía das Gatas formed a separate clade (Clade B) with Gorée Island, while other samples grouped into Clade A. This result corroborates previous findings by Lemer et al. (2014), who also observed significant separation of Gorée samples.

Long geographical distances and short larval durations in this species may contribute to the observed genetic structure (Nicolas et al., 1989; Wright et al., 2015). Additionally, sex-biased dispersion, where one sex disperses more, could further influence genetic structuring. According to Harrison, York & Young (2014), when sex-biased dispersion occurs in fragmented populations, genotypes of the more dispersive sex can be expected to be more randomly distributed among populations than genotypes of the other sex. Evidence of sex-biased dispersal has been documented in other marine molluscs, such as *Mytilus galloprovincialis* (Quesada, Skibinski & Skibinski, 1996) and *Cerastoderma glaucum* (Tarnowska et al., 2010), where male genotypes are more randomly distributed. The observed higher structuring in mtDNA compared to nDNA in Cabo Verde populations of *P. rudis* may suggest a similar phenomenon, with mitochondrial markers showing more pronounced differentiation (Dávalos & Russell, 2014; Yu et al., 2022).

Marine invertebrates with sessile adult stages often show patterns influenced by their larval dispersion capabilities (Butler, Vicente & de Gaulejac, 1993; Lee & Boulding, 2009). Some larvae spend relatively long periods in plankton and passive dispersion through ocean currents can allow sufficient gene flow to ensure relative genetic homogeneity within the geographic range of the species (Lee & Boulding, 2009). The high number of unique haplotypes in Cabo Verde suggests weak biogeographic affinity with the other archipelagos. Freitas et al. (2019) presented evidence for distinct biogeographic units of six marine groups with very different dispersal capacities within Macaronesia, with Cabo Verde forming a separate unit due to limited genetic exchange with other units (larvae, propagules, rafting adults, colonization events). This might be explained by the fact the Cabo Verde archipelago is situated within the Sahelian upwelling ecoregion in a single biogeographic unit, the West African Transition Province. Although the tendency for planktotrophic species is to show less or no genetic divergence at the regional level, a few cases of population structure have been reported for species with planktonic larvae. Sanna et al. (2013) showed the existence of population under-structuring in *P. nobilis* in the Mediterranean Sea, due to the presence of hydrodynamic barriers, local currents and hydrographic isolation. Indeed, *P. rudis* demonstrates a notable population structure, possibly due to reduced genetic flow and limited larval dispersion.

Another factor to consider is the relatively low level of genetic erosion in *P. rudis* (Bijlsma & Loeschcke, 2012), due to the species limited human exploitation because of the low muscle mass and the low population density. Exploitation can also lead to decreased genetic differentiation between populations. When substantial portions of a population are harvested, the remaining individuals may not represent the full genetic scale of the original population. This can result in homogenized genetic characteristics, with fewer haplotypes and reduced differentiation, contrary to the findings in *P. rudis* (Gandra et al., 2020). Indeed, in the sister species *P. nobilis*, higher mitochondrial variability was observed in Corsica-Sardinia, Elba Island, Sicily and the Lagoon of Venice, compared to populations in the Aegean and Tunisian coast. This suggests that human disturbance or bio-ecological changes may contribute to the loss of genetic variability (Sanna et al., 2024). The current study’s data are inconclusive as to supporting or rejecting these hypotheses.

Bayesian and maximum likelihood analysis of the same dataset showed a pattern remarkably similar to the haplotype network, providing support for the existence of two highly divergent clades in *P. rudis*, thus corroborating the findings of Lemer et al. (2014). Specifically, the sequences of *P. rudis* off the coast of Senegal and Baía das Gatas were distinctly and earlier separated from those of *P. carnea* and the remaining *P. rudis* populations from the Mediterranean, Atlantic Ocean (Canaries and Azores) and Cabo Verde. The average genetic distance between the two clades was considerable (6.4%) based on all two mitochondrial markers concatenated. For comparison, Wu et al. (2023) found that *Lepidodesma aligera* and *Lepidodesma languilati* were separated by an average genetic distance of only 4% based on COI marker.

According to Hart & Sunday (2007), the 95% parsimony connection limit, based on conventional DNA barcoding can be a useful tool for species discovery, potentially more effective than nuclear alleles. This limit appears to have a higher true-positive rate for discovering new cryptic species when applied to mtDNA. In contrast, frequent recombination between nuclear alleles may slow the rate at which ancestral polymorphisms shared among recently diverged species are lost from one (or both) through lineage sorting, thus reducing the rate at which haplotype differences between sister species approach the parsimony connection limit. Our data shows that *P. rudis* from Senegal and Baía das Gatas (São Vicente Island) may be a candidate species. This finding indicates previously unrecognized diversity within *P. rudis*, which may lead to a re-evaluation of the evolutionary relationships within the species. This has important implications for the taxonomy and conservation of these evolutionary units, which hold unique genetic heritage crucial to biodiversity preservation (Sanna et al., 2024). Further research should involve additional morphological and genetic data from the Baía das Gatas and Gorée populations to more accurately delineate species morphotypes.

Our study did not reveal significant morphological differences between the two clades of *P. rudis*. Similar findings were reported in other species, such as *P. saccata*, where morphological stasis was observed despite genetic differences (Lemer et al., 2014). Genetic information does not always align with morphological data. For example, analysis of the morphological and molecular differences between *Ruditapes philippinarum* (Adams & Reeve, 1850) and *Ruditapes decussatus* (Linnaeus, 1758) in the northeastern Adriatic Sea showed significant morphological similarities, with the species differentiation confirmed only by differences in the 16S rRNA gene analysis (Nerlović, Korlević & Mravinac, 2016). Conversely, morphological analysis of two *Corbicula fluminea* (Müller, 1774) populations in the Minho River estuary and the Lima River estuary (NW Portugal) showed significant shell shape differences, while genetic analysis revealed identical mtCOI sequence, indicating that both populations belong to *C. fluminea* (Sousa et al., 2007).

Environmental conditions can impose stabilizing selection in morphology, reducing or eliminating the morphological changes that might accompany speciation, since there are often few ways for an organism to adapt to harsh environments (Nevo, 2001). Consequently, speciation does not always involve morphological change (the morphological stasis). The true number of biological species is probably higher than the current count of nominal species, most of which are often outlined based solely on morphological characteristics (Bickford et al., 2007).

## Conclusion

This study highlights significant genetic divergence within *P. rudis*, revealing the potential cryptic speciation, particularly in the populations of the coast of Senegal and Baía das Gatas. Despite the limited sample size, the results reveal significant genetic divergence, suggesting the possible existence of a previously unrecognized species. The minimal morphological variation alongside this genetic differentiation highlights the limitations of relying only on traditional taxonomy and emphasizes the value of molecular data in species identification and delimitation. Additionally, the study demonstrates the role of human disturbance in shaping genetic diversity, showing higher mitochondrial variability in regions less impacted by human activities, as the population from Santa Luzia with larger and, consequently, older samples. On the other hand, the sample group from Baía das Gatas is likely to be under more human disturbance. Hence there is a need for more extensive sampling, in number and space, and deeper investigation into the evolutionary relationships within the genus *Pinna*. Understanding these dynamics is critical for developing effective conservation strategies to protect their unique genetic units, which are essential for maintaining biodiversity in these marine ecosystems.

## Supplemental Information

10.7717/peerj.18328/supp-1 Supplemental Information 1 DNA sequences of the different markers used in this study.

10.7717/peerj.18328/supp-2 Supplemental Information 2 Figure S1. Phylogenetic relationships of *Pinna* species inferred from the combined Maximum Likelihood and Baesian analysis of two mitochondrial markers (COI and 16S rRNA). Tree topology was obtained from Maximum Likelihood analysis. Samples from Cabo Verde are in blue. The two clades of *Pinna rudis* form Cabo Verde (A and B) are indicated by labelled bars. Scale bar represents 0.4 substitutions per site. The bootstrap values for ML and BA appear one after the other at each node, respectively. The *Streptopinna sacata* sequences were used as outgroups.

10.7717/peerj.18328/supp-3 Supplemental Information 3 Figure S2. Phylogenetic relationships of *Pinna* species inferred from the combined Maximum Likelihood and Bayesian analysis of 28S rRNA. Tree topology was obtained from Maximum Likelihood analysis. Samples from Cabo Verde are in blue. Scale bar represents 0.004 substitutions per site. The bootstrap values for ML and BA appear one after the other at each node, respectively. The *Streptopinna sacata* sequences were used as outgroups.

10.7717/peerj.18328/supp-4 Supplemental Information 4 Table S1. Raw Data - Biometric data obtained from 5 parameters in a total of 32 shell samples. Legend: L- Maximum shell length, LT- Maximum shell width, W - Total shell weight; Fraction of total length by shell weight - L / W, Fraction of total width by shell weight - Lt / W.

10.7717/peerj.18328/supp-5 Supplemental Information 5 Table S2. Haplotype frequency distributions of COI *P. rudis* samples

10.7717/peerj.18328/supp-6 Supplemental Information 6 Table S3. Haplotype frequency distributions of 16S RNA *P. rudis* samples

10.7717/peerj.18328/supp-7 Supplemental Information 7 Table S4. Haplotype frequency distributions of 28S RNA *P. rudis* samples

## Additional Information and Declarations

### Data Availability

The following information was supplied regarding data availability: The sequences are available at GenBank: OQ055700–OQ055719 (16S rRNA); OQ026776–OQ026795 (COI); OQ026797–OQ026816 (28S rRNA). The other raw data are available in the Supplemental Files.
