# Supplementary material for: Genetic analysis of Pinna rudis L 1758 (Mollusca, Bivalvia, Pinnidae) in the Northwest Cabo Verde Islands (Central-East Atlantic)
Source: PeerJ. 2025 Jan 8;13:e18328. doi: 10.7717/peerj.18328 (PMC11724654; doi:10.7717/peerj.18328)
Supplement: Supplemental Information 4 — Raw Data - Biometric data obtained from 5 parameters in a total of 32 shell samples. Legend: L- Maximum shell length, LT- Maximum shell width, W - Total shell weight; Fraction of total length by shell weight - L / W, Fraction of total width by shell weight - Lt / W. [file peerj-13-18328-s004.docx]

## Table S1

**Manuscript Title**

Genetic structure of *Pinna rudis* L. 1758 (Mollusca, Bivalvia, Pinnidae) in the Cabo Verde Islands (Central-East Atlantic)

**Authors**

Evandro Pires Lopes ^1, 2, 3^ Sarah Sofia Dos Santos ^1^, Raquel Xavier ^2, 3^

Joana L Santos ^2, 3^, M Pilar Cabezas ^4,5^, Fernando Sequeira ^2, 3^, António Múrias dos Santos ^2, 3^

**Affiliations**

^1^ Instituto de Engenharias e Ciências do Mar, Universidade Técnica do Atlântico, CP 163 São Vicente, Cabo Verde

^2^ CIBIO, Centro de Investigação em Biodiversidade e Recursos Genéticos, InBIO Laboratório Associado, Campus de Vairão, Universidade Do Porto, 4485-661, Vairão, Portugal

^3^ BIOPOLIS Program in Genomics, Biodiversity and Land Planning, CIBIO, Campus de Vairão, 4485-661, Vairão, Portugal

^4^ Centre of Molecular and Environmental Biology (CBMA) and ARNET-Aquatic Research Network, Department of Biology, University of Minho, Campus de Gualtar, 4710-057 Braga, Portugal
^5^ Institute of Science and Innovation for Bio-Sustainability (IB-S), University of Minho, Campus de Gualtar, 4710-057 Braga, Portugal

**Corresponding author:**

Evandro P. Lopes

Instituto de Engenharias e Ciências do Mar

Universidade Técnica do Atlântico

CP 163, São Vicente, Cabo Verde

Email: elopes@uta.cv

Phone: +238 9506462; +238 2321113

Table S1. Biometric data obtained from 5 parameters in a total of 32 shell samples. Legend: L- Maximum shell length, LT- Maximum shell width, W - Total shell weight; Fraction of total length by shell weight - L / W, Fraction of total width by shell weight - Lt / W.

| Samples ID | Island | Sample place | GPS | L | LT | W | LT/W | L/W |
| --- | --- | --- | --- | --- | --- | --- | --- | --- |
| 1 | Santo Antão | Porto Novo | 17° 0'4604"N / 25° 5'579"W | 183,00 | 91,00 | 57,45 | 1,58 | 3,19 |
| 2 |  |  |  | 188,00 | 93,95 | 42,82 | 2,19 | 4,39 |
| 3 |  |  |  | 128,50 | 65,80 | 18,23 | 3,61 | 7,05 |
| 4 |  |  |  | 177,50 | 82,10 | 35,00 | 2,35 | 5,07 |
| 5 |  |  |  | 150,50 | 91,40 | 35,31 | 2,59 | 4,26 |
| 6 |  |  |  | 124,50 | 65,85 | 17,06 | 3,86 | 7,30 |
| 7 | São Vicente | Laginha | 16°53'5506"N / 24°59'3634"W | 168,00 | 72,85 | 36,27 | 2,01 | 4,63 |
| 8 |  |  |  | 159,00 | 70,70 | 24,32 | 2,91 | 6,54 |
| 9 |  |  |  | 138,00 | 75,25 | 26,56 | 2,83 | 5,20 |
| 10 |  |  |  | 180,50 | 88,07 | 61,71 | 1,43 | 2,92 |
| 11 |  |  |  | 169,00 | 83,10 | 66,95 | 1,24 | 2,52 |
| 12 |  |  |  | 152,50 | 64,55 | 32,55 | 1,98 | 4,69 |
| 13 |  |  |  | 178,00 | 78,35 | 52,41 | 1,49 | 3,40 |
| 14 |  |  |  | 166,50 | 73,45 | 57,78 | 1,27 | 2,88 |
| 15 |  |  |  | 135,00 | 80,80 | 23,40 | 3,45 | 5,77 |
| 16 |  |  |  | 136,00 | 80,80 | 43,97 | 1,84 | 3,09 |
| 17 |  |  |  | 156,00 | 77,40 | 32,11 | 2,41 | 4,86 |
| 18 |  |  |  | 186,00 | 112,70 | 67,50 | 1,67 | 2,76 |
| 19 |  |  |  | 161,50 | 68,30 | 44,74 | 1,53 | 3,61 |
| 20 |  |  |  | 202,50 | 87,55 | 54,60 | 1,60 | 3,71 |
| 21 |  |  |  | 163,00 | 63,90 | 45,02 | 1,42 | 3,62 |
| 22 |  |  |  | 158,00 | 91,30 | 30,04 | 3,04 | 5,26 |
| 23 | São Vicente | Baía das Gatas | 16°54'755"N / 24°54'2168"W | 185,00 | 92,40 | 28,95 | 3,19 | 6,39 |
| 24 |  |  |  | 192,50 | 78,00 | 22,30 | 3,50 | 8,63 |
| 25 |  |  |  | 135,50 | 69,75 | 24,40 | 2,86 | 5,55 |
| 26 |  |  |  | 166,00 | 85,80 | 70,95 | 1,21 | 2,34 |
| 27 |  |  |  | 222,50 | 98,65 | 41,72 | 2,36 | 5,33 |
| 28 |  |  |  | 183,00 | 102,95 | 41,53 | 2,48 | 4,41 |
| 29 |  |  |  | 157,50 | 69,55 | 29,78 | 2,34 | 5,29 |
| 30 | Sta. Luzia | Portinho | 16°45'945"N / 24°45'3073"W | 309,00 | 147,30 | 131,68 | 0,44 | 0,93 |
| 31 |  |  |  | 226,00 | 127,70 | 113,84 | 1,12 | 1,99 |
| 32 |  |  |  | 227,50 | 106,45 | 103,99 | 1,02 | 2,19 |
