# Supplementary material for: Genetic analysis of Pinna rudis L 1758 (Mollusca, Bivalvia, Pinnidae) in the Northwest Cabo Verde Islands (Central-East Atlantic)
Source: PeerJ. 2025 Jan 8;13:e18328. doi: 10.7717/peerj.18328 (PMC11724654; doi:10.7717/peerj.18328)
Supplement: Supplemental Information 6 — Haplotype frequency distributions of 16S RNA P. rudis samples [file peerj-13-18328-s006.docx]

## Table S3

**Manuscript Title**

Genetic structure of *Pinna rudis* L. 1758 (Mollusca, Bivalvia, Pinnidae) in the Cabo Verde Islands (Central-East Atlantic)

**Authors**

Evandro Pires Lopes ^1, 2, 3^ Sarah Sofia Dos Santos ^1^, Raquel Xavier ^2, 3^

Joana L Santos ^2, 3^, M Pilar Cabezas ^4,5^, Fernando Sequeira ^2, 3^, António Múrias dos Santos ^2, 3^

**Affiliations**

^1^ Instituto de Engenharias e Ciências do Mar, Universidade Técnica do Atlântico, CP 163 São Vicente, Cabo Verde

^2^ CIBIO, Centro de Investigação em Biodiversidade e Recursos Genéticos, InBIO Laboratório Associado, Campus de Vairão, Universidade Do Porto, 4485-661, Vairão, Portugal

^3^ BIOPOLIS Program in Genomics, Biodiversity and Land Planning, CIBIO, Campus de Vairão, 4485-661, Vairão, Portugal

^4^ Centre of Molecular and Environmental Biology (CBMA) and ARNET-Aquatic Research Network, Department of Biology, University of Minho, Campus de Gualtar, 4710-057 Braga, Portugal
^5^ Institute of Science and Innovation for Bio-Sustainability (IB-S), University of Minho, Campus de Gualtar, 4710-057 Braga, Portugal

**Corresponding author:**

Evandro P. Lopes

Instituto de Engenharias e Ciências do Mar

Universidade Técnica do Atlântico

CP 163, São Vicente, Cabo Verde

Email: elopes@uta.cv

Phone: +238 9506462; +238 2321113

Table S3. Haplotype frequency distributions of 16S RNA *P. rudis* samples

| **Region/population** | | **16S RNA** | | | | | | | | | | | | | |
| --- | --- | --- | --- | --- | --- | --- | --- | --- | --- | --- | --- | --- | --- | --- | --- |
|  |  | *h1* | *h2* | *h3* | *h4* | *h5* | *h6* | *h7* | *h8* | *h9* | *h10* | *h11* | *h12* | *h13* | *h14* |
| CV - Santo Antão | Porto Novo | 3 | 3 |  |  |  |  |  |  |  |  |  |  |  |  |
| CV -São Vicente | Laginha | 1 | 2 | 2 | 1 |  |  |  |  |  |  |  |  |  |  |
|  | Baía das Gatas | 1 | 1 |  |  | 2 | 1 |  |  |  |  |  |  |  |  |
| CV -Santa Luzia | Portinho |  |  |  |  |  |  | 2 | 1 |  |  |  |  |  |  |
| Azores | São Miguel |  |  |  |  |  |  |  |  | 1 |  | 1 |  | 1 |  |
| Canarias island | El Hierro |  |  |  |  |  |  |  |  |  |  | 1 | 1 | 1 |  |
|  | Tenerife |  |  |  |  |  |  |  |  |  |  | 2 | 1 |  |  |
|  | Fuerteventura |  |  |  |  |  |  |  |  |  |  |  |  | 1 |  |
| Senegal | Gorée Island |  |  |  |  |  |  |  |  |  |  |  |  |  | 1 |
| Spain | Murcia |  |  |  |  |  |  |  |  | 1 |  |  |  |  |  |
|  | Columbrete Island |  |  |  |  |  |  |  |  |  | 1 |  |  |  |  |
| ***Total*** | | **5** | **6** | **2** | **1** | **2** | **1** | **2** | **1** | **2** | **1** | **4** | **2** | **3** | **1** |
